# Predicting potential microbe–disease associations based on dual branch graph convolutional network

**DOI:** 10.1111/jcmm.18571

**Published:** 2024-07-31

**Authors:** Jing Chen, Yongjun Zhu, Qun Yuan

**Affiliations:** ^1^ School of Electronic and Information Engineering Suzhou University of Science and Technology Suzhou China; ^2^ Department of Respiratory Medicine, The Affiliated Suzhou Hospital of Nanjing University Medical School Suzhou China

**Keywords:** association prediction, disease, dual branch graph convolutional network, microbe, random walk with restart

## Abstract

Studying the association between microbes and diseases not only aids in the prevention and diagnosis of diseases, but also provides crucial theoretical support for new drug development and personalized treatment. Due to the time‐consuming and costly nature of laboratory‐based biological tests to confirm the relationship between microbes and diseases, there is an urgent need for innovative computational frameworks to anticipate new associations between microbes and diseases. Here, we propose a novel computational approach based on a dual branch graph convolutional network (GCN) module, abbreviated as DBGCNMDA, for identifying microbe–disease associations. First, DBGCNMDA calculates the similarity matrix of diseases and microbes by integrating functional similarity and Gaussian association spectrum kernel (GAPK) similarity. Then, semantic information from different biological networks is extracted by two GCN modules from different perspectives. Finally, the scores of microbe–disease associations are predicted based on the extracted features. The main innovation of this method lies in the use of two types of information for microbe/disease similarity assessment. Additionally, we extend the disease nodes to address the issue of insufficient features due to low data dimensionality. We optimize the connectivity between the homogeneous entities using random walk with restart (RWR), and then use the optimized similarity matrix as the initial feature matrix. In terms of network understanding, we design a dual branch GCN module, namely GlobalGCN and LocalGCN, to fine‐tune node representations by introducing side information, including homologous neighbour nodes. We evaluate the accuracy of the DBGCNMDA model using five‐fold cross‐validation (5‐fold‐CV) technique. The results show that the area under the receiver operating characteristic curve (AUC) and area under the precision versus recall curve (AUPR) of the DBGCNMDA model in the 5‐fold‐CV are 0.9559 and 0.9630, respectively. The results from the case studies using published experimental data confirm a significant number of predicted associations, indicating that DBGCNMDA is an effective tool for predicting potential microbe–disease associations.

## 1 INTRODUCTION

Microbes refer to the smallest biological entities, mainly including bacteria, fungi, viruses and other diverse groups.^1^, ^2^ They are widely distributed in various environments on Earth, such as soil, water, air and the surfaces of organisms.^3^, ^4^ In human life and health, microbes play significant roles, contributing to the maintenance of ecological balance both inside and outside the human body while also potentially causing diseases.^5^, ^6^, ^7^ For instance, certain bacteria, fungi and viruses can lead to infectious diseases such as influenza, pneumonia and dysentery,^8^, ^9^ while other microbes facilitate food fermentation, producing beneficial lactobacilli and yeast.^10^, ^11^ Additionally, microbes form symbiotic relationships with various parts of the human body, including the digestive tract, skin and respiratory tract, exerting indispensable effects on human health.^12^, ^13^, ^14^

With the continuous development of biotechnology, research on microbes has become increasingly profound. Analysis and comparison of microbial communities can reveal the diversity, composition and functional characteristics of microbes in different environments, providing important clues for understanding the relationship between microbes and diseases.^15^, ^16^ For example, the abnormal increase or decrease of certain microbes may be closely related to the occurrence and development of certain diseases, such as the link between imbalanced gut microbiota and intestinal diseases,^17^, ^18^, ^19^, ^20^ and the occurrence of skin diseases associated with changes in skin microbiota.^21^, ^22^ Therefore, a comprehensive understanding of the ecological, genetic and physiological characteristics of microbes can help predict changes in microbial communities associated with diseases, providing theoretical guidance and technical support for early prevention, accurate diagnosis and effective treatment of diseases.^23^, ^24^

The relationship between microbes and diseases is intricate and multifaceted, involving deep‐seated mechanisms such as the biological characteristics of microbes, host immune responses and the influence of external environmental factors.^25^, ^26^, ^27^ Firstly, many microbes directly induce diseases by invading human tissues or releasing toxins, such as bacterial pneumonia and viral influenza.^28^, ^29^, ^30^ The pathogenicity of these microbes depends on their virulence factors, invasiveness and ability to evade host immune responses. Secondly, microbes can trigger diseases by influencing the host's immune system.^31^ Some microbes activate the host's immune response, leading to excessive inflammation and tissue damage, such as rheumatic fever caused by *Streptococcus* ^32^, ^33^; while others suppress the host's immune response, resulting in immune suppression and chronic infections, such as AIDS caused by HIV.^34^, ^35^ Additionally, the ecological balance of microbes plays a crucial role both inside and outside the human body.^36^, ^37^, ^38^ Disruption of this balance by any factor may lead to the occurrence of diseases. For example, excessive use of antibiotics may disrupt the balance of intestinal flora, leading to intestinal diseases.^38^, ^39^, ^40^ Therefore, a comprehensive understanding of the relationship between microbes and diseases not only helps in the prevention, diagnosis and treatment of related diseases but also provides an important theoretical basis for the development of more effective treatment strategies.

Research on the relationship between microbes and diseases holds significant importance and profound implications. First, understanding the association between microbes and diseases can aid in better disease prevention.^41^, ^42^, ^43^ By understanding the pathogenic mechanisms of specific microbes, preventive strategies such as vaccines and antimicrobial drugs can be developed, thereby reducing the incidence of diseases.^44^, ^45^ Second, awareness of the association between microbes and diseases contributes to improving the early diagnosis rate of diseases. Microbes may exhibit specific biomarkers or biological characteristics at different stages of disease development.^46^, ^47^, ^48^ By detecting these biomarkers, early diagnosis of diseases can be achieved, enhancing treatment effectiveness and prognosis. Moreover, in‐depth understanding of the regulatory role of microbes on the host immune system can provide new insights and approaches for immune modulation therapy and personalized medicine. Lastly, studying the relationship between microbes and diseases also facilitates the discovery of new therapeutic targets and drugs. Understanding the mechanisms of action of microbes in disease occurrence and progression can provide a theoretical basis for developing targeted therapeutic drugs against microbes, offering new treatment strategies for combating drug‐resistant microbial infections and chronic infections.^49^ Research on the association between microbes and diseases not only contributes to disease prevention and diagnosis but also provides important theoretical support for drug development and personalized treatment, thereby contributing to the improvement of human health and medical standards.

Conventional methods for discovering the association between microbes and diseases include direct pathogen detection, microbiome studies and host–microbe interaction network analysis. Direct pathogen detection confirms the association with diseases by detecting pathogenic microbes in patient samples^50^; microbiome studies utilize high‐throughput sequencing technologies to compare microbial composition differences between patients and healthy control groups^51^; while host–microbe interaction network analysis constructs interaction networks by integrating host and microbiome data.^52^ However, these methods also have some limitations. For instance, direct pathogen detection may overlook some potential microbial pathogenic factors, microbiome studies are limited by sample quantity and quality, and the complexity of host–microbe interaction network analysis may result in difficulties in interpreting the results.

With the rapid development of bioinformatics and life science technologies, a large amount of biomedical data has been accumulated. Based on this, researchers have developed various computational methods to discover potential associations between human microbes, drugs and diseases.^14^, ^53^, ^54^, ^55^, ^56^, ^57^ According to different prediction methods, these methods can be divided into the following categories: path‐based methods, binary local models, ensemble learning and random walk methods. Path‐based methods typically measure the weight of potential paths as part of unknown associations by considering indirect paths in the network. The KATZHMDA model developed by Chen et al.^58^ predicts new microbial disease associations on a scale by combining known microbial disease associations and Gaussian interaction spectral kernel similarity between microbes and diseases. A typical ensemble learning based method is the ABHMDA prediction model, which uses adaptive boosting for ensemble learning. In Peng et al.'s study, decision trees were selected as weak classifiers in the development process of ABHMDA prediction models. This model can be applied to new diseases without any known associated microbes. The binary local model evaluates the correlation between microbes and diseases by analysing the two dimensions of disease and microbes, and calculates the predicted score.^59^, ^60^ An innovative computational model, LRLSHMDA, developed by Wang et al., designed two objective functions for microbes and diseases, and minimized these two functions through Laplace regularization.^61^ This model combines Gaussian interaction profile kernel similarity measure and Laplacian regularized least squares (LapRLS) classification technique, effectively utilizing the structural information in known microbial disease association networks, including potential data of vertices and edges. Despite providing satisfactory results, existing predictive factors have not fully utilized the structural semantic information of biological networks to effectively learn complex association patterns in graph structured data. Therefore, in order to process these data more effectively, GCNs have been proposed and successfully applied to various tasks in bioinformatics, such as disease gene association detection, drug target interaction prediction and drug repositioning.^62^, ^63^, ^64^, ^65^

Neural networks excel at capturing local spatial patterns through convolutional modules.^66^, ^67^ Inspired by the ability of GCNs to effectively capture nonlinear association patterns in complex networks,^68^, ^69^, ^70^, ^71^, ^72^, ^73^, ^74^ we propose a novel computational method called DBGCNMDA for identifying microbe–disease associations. Organizing the associations between microbes and diseases as a network, where microbes or diseases are modelled as vertices and associations are regarded as edges, DBGCNMDA is designed from two different perspectives of the perception field of GCN modules to capture rich semantic information from diverse biological networks. Specifically, the GlobalGCN module is employed to learn representations of nodes in the microbe–disease association network, where microbial node features are learned from associated disease nodes and disease node features are learned from associated microbial nodes. On the other hand, the LocalGCN module is utilized to further learn representations of nodes in two homogeneous similar networks, with microbial node representations obtained from microbial neighbourhood information and disease node representations obtained similarly. Finally, we treat the problem as a link prediction task and predict microbe–disease association scores based on the learned features. Experimental results demonstrate that DBGCNMDA outperforms other state‐of‐the‐art methods in terms of performances.

Overall, our main contributions are concluded as follows:

1. We have calculated the similarity matrix of diseases and microbes by integrating functional similarity and GAPK similarity.
2. We extend the disease nodes to address the issue of insufficient features due to low data dimensionality.
3. We optimize the connectivity between the homogeneous entities using RWR, and then use the optimized similarity matrix as the initial feature matrix.
4. We extract semantic information from different biological networks through two differently perspective GCN modules, namely GlobalGCN and LocalGCN, to fine‐tune node representations by introducing side information, including homologous neighbour nodes.

## 2 METHODS AND MATERIALS

### 2.1 Datasets

We utilized the Human Microbe‐Disease Association Database (HMDAD; http://www.cuilab.cn/hmdad) for MDA prediction, which encompasses 450 MDAs between 292 microbes and 39 diseases.^75^ The dataset $D_{\mathrm{all}}$ consists of a benchmark set $D_{\text{benchmark}}$ and an independent test set $D_{\text{test}}$. $D_{\mathrm{all}}^{+}$ denotes the positive set containing 450 positive associations, while $D_{\mathrm{all}}^{-}$ represents the negative set containing 450 negative associations. The benchmark set $D_{\text{benchmark}}$ was randomly divided into five subsets, where four subsets served as training set $D_{\text{train}}$, and the remaining subset was utilized as the validation set $D_{\text{validation}}$. Through 5‐fold CV, the hyper parameters of the method were optimized on the validation set. Finally, the model was evaluated on the independent test set $D_{\text{test}}$ and compared with other relevant methods.

### 2.2 DBGCNMDA

**FIGURE 1:**
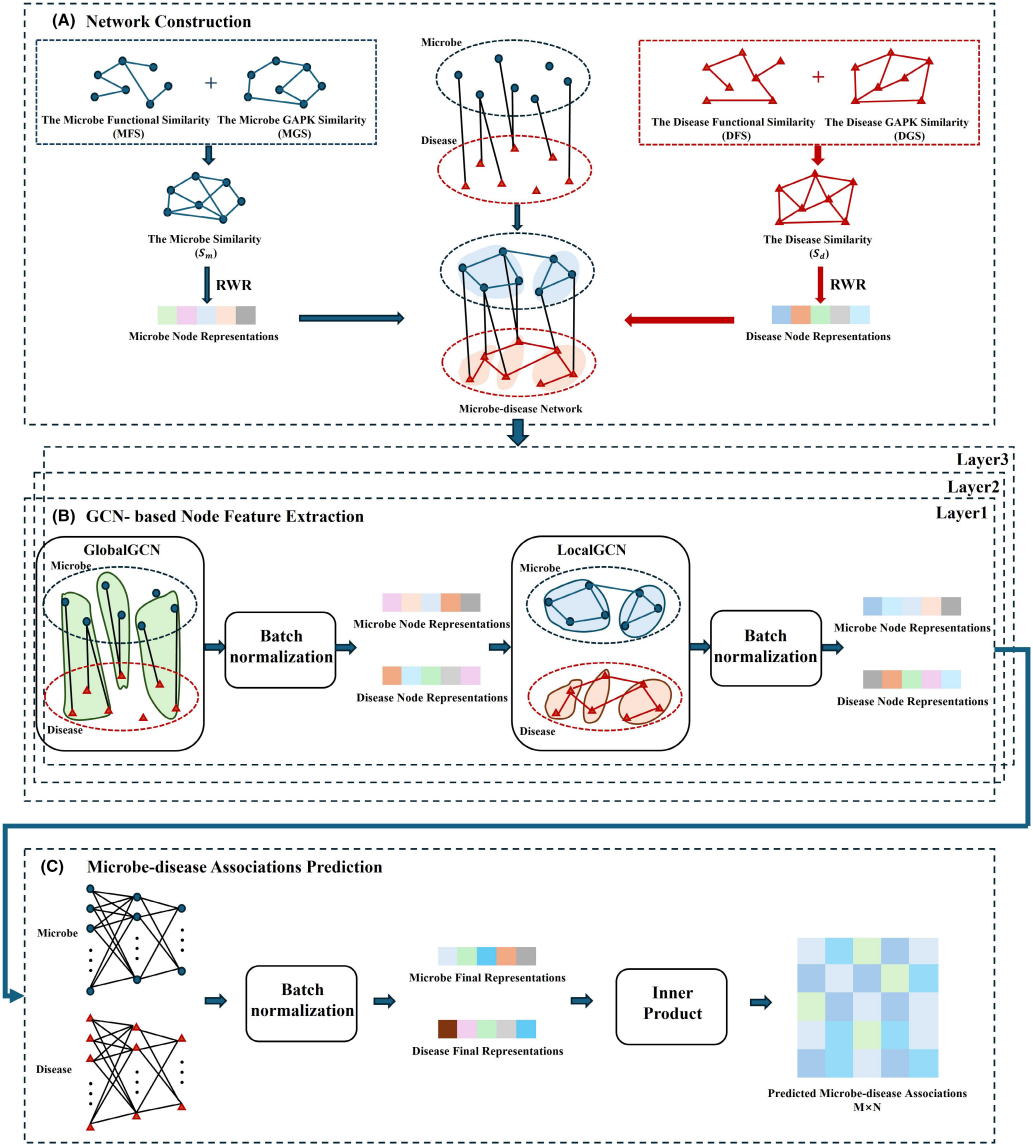
Flowchart of potential microbe–disease association prediction based on DBGCNMDA.

In this section, we propose a prediction model, DBGCNMDA, based on a dual branch graph neural network to forecast the association between diseases and microbes. The framework of DBGCNMDA is depicted in Figure 1 and primarily consists of three steps: heterogeneous network construction (Figure 1A), node feature extraction based on GCN (Figure 1B), and prediction of microbe–disease associations (Figure 1C). First, DBGCNMDA computes the similarity matrices of diseases and microbes by integrating their functional similarity and Gaussian interaction profile kernel (GAPK) similarity. Then, semantic information from different biological networks is extracted through two different perception field of GCN modules. Finally, based on the extracted features, DBGCNMDA predicts the scores of microbe–disease associations.

### 2.3 Network construction

#### 2.3.1 Edge representation

In the constructed microbe–disease network, three types of edges are utilized to represent the relationships between different nodes. Between two heterogeneous nodes, edges are employed to depict the original interactions among *m* microbes (*m* = 292) and *n* diseases (*n* = 39). In the following equation, the adjacency matrix representing the microbe–disease associations is denoted as $A_{\mathrm{md}}$, where if there is experimentally validated correlation between the *i*‐th microbe and the *j*‐th disease, $A_{i,j}=1$; otherwise, $A_{i,j}=0$.

(1) $$A_{i,j}=\left\{\begin{array}{ll} 1, & \text{microbe}\;m_{i}\;\text{associates with disease}\;n_{j}\\ 0, & \text{else} \end{array}\right.$$

Two other types of edges, referred to as ‘similar edges’ are included in the similarity subnetworks, indicating the similarity between two homogeneous nodes and calculated based on information from microbe–microbe and disease–disease biological entities. Kamneva et al.^76^ devised the ‘microbe–microbe functional association index’ to capture interactions between proteins encoded by genomes of two microbes. We employed a similar approach to compute microbe functional similarity. We retrieved the protein–protein functional interaction network from the STRING v11 database (https://stringdb.org). For further details on microbe functional similarity computation, please refer to.^77^ We denote MFS to represent the functional similarity between microbes, and then compute the functional similarity matrix among *m* microbes, where ${\mathrm{MFS}}_{\left(m_{i},m_{j}\right)}$ denotes the similarity between microbe $m_{i}$ and $m_{j}$. Based on the assumption that similar diseases tend to interact with similar genes,^78^, ^79^ we calculate the functional similarity of diseases based on the functional associations among disease‐related genes. We employ the disease functional similarity assessment method proposed by Long et al. to compute the functional similarity matrix DFS among *n* diseases,^80^ where ${\mathrm{DFS}}_{\left(n_{i},n_{j}\right)}$ represents the similarity between two diseases $n_{i}$ and $n_{j}$.

The GAPK function is a radially symmetric function that exhibits good clustering effects for examples with linearly separable forms.^81^ Similar to the work of Peng et al., we denote $V_{m_{i}}$ (the *i*‐th row of *Y*) and $V_{m_{j}}$ (the *j*‐th row of *Y*) as representing two microbes $m_{i}$ and $m_{j}$, respectively.^82^ Their similarity can be computed as follows:

(2) $${\mathrm{MGS}}_{\left(m_{i},m_{j}\right)}=\exp\left(-\Theta_{m}{\left\|V_{m_{i}}-V_{m_{j}}\right\|}^{2}\right)$$

where

(3) $$\Theta_{m}=\frac{1}{m}\sum_{i=1}^{m}{\left\|V_{m_{i}}\right\|}^{2}$$

Similarly, we calculate the disease GAPK similarity DGS.

To more accurately assess the similarity of microbes/diseases, we evaluate the functional similarity of microbes/diseases from biological characteristics and assess the GAPK similarity of microbes/diseases from the network topology structure. To combine functional similarity and GAPK similarity, we use two types of information for microbe/disease similarity assessment to improve MDA recognition performance. By integrating their functional similarity and GAPK similarity, we compute the final microbe similarity matrix $S_{m}$ as follows:

(4) $$S_{m}=\left\{\begin{array}{ll} \frac{{\mathrm{MFS}}_{\left(m_{i},m_{j}\right)}+{\mathrm{MGS}}_{\left(m_{i},m_{j}\right)}}{2}, & \text{if}\;{\mathrm{MFS}}_{\left(m_{i},m_{j}\right)}\neq 0\\ {\mathrm{MGS}}_{\left(m_{i},m_{j}\right)}, & \text{else} \end{array}\right.$$

Similarly, disease similarity matrix $S_{d}$ is computed by Equation (5) as follows:

(5) $$S_{d}=\left\{\begin{array}{ll} \frac{{\mathrm{DFS}}_{\left(n_{i},n_{j}\right)}+{\mathrm{DGS}}_{\left(n_{i},n_{j}\right)}}{2}, & \text{if}\;{\mathrm{DFS}}_{\left(n_{i},n_{j}\right)}\neq 0\\ {\mathrm{DGS}}_{\left(n_{i},n_{j}\right)}, & \text{else} \end{array}\right.$$

#### 2.3.2 Node representation

In the constructed heterogeneous microbe–disease association network, two types of nodes are used to represent microbes and diseases, respectively. In this study, the connectivity relationship between homogeneous entities was optimized using RWR, especially for non‐neighbour nodes and high‐order nodes, and the optimized similarity matrix was used as the initial feature matrix.^83^ Using a combined microbe and disease similarity matrix as input for RWR. Obtain initial node features by considering the global topology information of each network. The initial node representation of microbes generated by RWR is calculated using the following formula:

(6) $$M_{i,j}^{k+1}\left(i\right)=\left(1-\gamma \right)e_{i,j}+\gamma M_{i,j}^{k}\left(i\right)S_{m}\left(m_{i},m_{j}\right)$$

(7) $$M\left(i\right)=\left[M_{i,1}^{\infty }\left(i\right),M_{i,2}^{\infty }\left(i\right),\ldots ,M_{i,j}^{\infty }\left(i\right),\ldots ,M_{i,m}^{\infty }\left(i\right)\right]$$

where $M_{i,j}^{k}$ represents the probability of walking from microbe node $m_{i}$ to node $m_{j}$ after k hops. $e_{i,j}$ represents the initial probability of walking from the microbe node $m_{i}$ to node $m_{j}$, and *e* is the identity matrix. $S_{m}\left(m_{i},m_{j}\right)$ is the transition probability obtained from the similarity matrix $S_{m}$, and γ is the restart probability. Combine the probabilities associated with $m_{i}$ all other microbe nodes to generate a node representing $M\left(i\right)$ for microbe $m_{i}$. Similarly, the initial node representation $D\left(i\right)$ of a disease can be calculated as follows:

(8) $$D_{i,j}^{k+1}\left(i\right)=\left(1-\gamma \right)e_{i,j}+\gamma D_{i,j}^{k}\left(i\right)S_{d}\left(d_{i},d_{j}\right)$$

(9) $$D\left(i\right)=\left[D_{i,1}^{\infty }\left(i\right),D_{i,2}^{\infty }\left(i\right),\ldots ,D_{i,j}^{\infty }\left(i\right),\ldots ,D_{i,n}^{\infty }\left(i\right)\right]$$

In order to solve the problem of insufficient features caused by low data dimensions, we extended the disease nodes by increasing the feature dimension of each disease node from 39 to 1000. Specifically, we generated polynomial features using the original input features, which can better reflect the interactions of different features in different dimensions. Polynomial features refer to combinations that limit the degree of features to a specified degree or less.

### 2.4 GCN‐based node feature extraction

Graph convolutional network (GCN) plays an important role in identifying the association between microbes and diseases. GCN has the ability to aggregate information from adjacent nodes and capture potential network structures, thereby effectively extracting discriminative node features. The proposed model utilizes GCN to learn the characteristics of microbe and disease nodes, which can better understand the structure of heterogeneous microbe–disease association networks.

Each GCN layer updates nodes to generate new node embeddings. In each layer, the embedded representation of nodes updates as GCN information propagates and aggregates. The node embedding $E^{k}$ of layer *k* is updated by the *k*‐th GCN layer based on the node embedding $E^{k-1}$ of layer (*k*−1)‐th GCN using the following formula:

(10) $$E^{k}=\delta \left({\tilde{D}}^{-\frac{1}{2}}{\tilde{S}\tilde{D}}^{-\frac{1}{2}}E^{k-1}W^{k-1}\right)$$

(11) $$\tilde{S}=I+S$$

(12) $${\tilde{D}}_{i,i}=\sum_{j}{\tilde{S}}_{i,j}$$

where *S* represents the adjacency matrix representing the relationships between all nodes in the network, and *I* represents the identity matrix. $\tilde{D}$ represents the degree matrix of $\tilde{S}$, $W^{k-1}$ represents the trainable parameter matrix of the GCN model, $\delta \left(\cdot \right)$ is a nonlinear activation function.

Using GCN to extract node information from heterogeneous microbe disease graphs, node representations are only learned from their heterogeneous neighbouring nodes. However, the association between microbes and diseases is too sparse to provide sufficient information for GCN to capture differential representations. Therefore, two key modules, GlobalGCN and LocalGCN are designed to fine tune node representations by introducing side information, including isomorphic neighbour nodes. First, in GlobalGCN, we aggregated node information in the microbe–disease interaction network. The characteristics of microbe nodes are obtained from adjacent disease node information, and vice versa. Second, we utilize LocalGCN to further capture semantic information from two isomorphic and similar networks. The node representation obtained by GlobalGCN is used as the initial node feature for the LocalGCN module. The constructed microbe–microbe similarity network and disease–disease similarity network are the two main inputs of LocalGCN. Generate microbe node representations by capturing neighbouring microbe information, and learn disease node representations from neighbouring disease information.

### 2.5 Association prediction for microbes and diseases

The fully connected layer can effectively reduce redundancy and noise and extract more useful feature representations through feature combination, nonlinear mapping, dimensionality reduction compression and regularization. Our model designs three continuous fully connected layers to extract advanced node features.

The microbe nodes and disease nodes extracted from the GCN module are represented as $R_{m_{i}}$ and $R_{d_{j}}$, respectively. After intensive computation, the final microbial and disease node representations are obtained as $R_{m_{i}}^{\prime }$ and $R_{d_{j}}^{\prime }$, respectively. The correlation score $\tilde{A}$ between the final microbe $m_{i}$ and the disease $d_{j}$ can be calculated using the following formula:

(13) $${\tilde{A}}_{i,j}=R_{m_{i}}^{\prime }{R^{\prime }}_{d_{j}}^{T}$$

where $\tilde{A}$ is the final predicted score matrix. The higher the elements ${\tilde{A}}_{i,j}$, the more likely microbe $m_{i}$ is to be associated with disease $d_{j}$.

Loss function uses mean square error to minimize the Frobenius norm of the difference between the final predicted score matrix $\tilde{A}$ and the label matrix *A*. However, the number of negative associations far exceeds that of positive associations. To address the balance in training samples, an α‐Enhanced loss function^84^ that emphasis positive sample learning is employed, formulated as follows:

(14) $$\text{Loss}={\left\|A^{\prime }-\tilde{A}\right\|}_{F}^{2}+\mu {\left\|W\right\|}_{2}^{2}$$

where

(15) $$A^{\prime }=\left\{\begin{array}{ll} 0, & \text{if}\;A_{i,j}=0\;\text{or}\;A_{i,j}\in D_{\text{test}}\\ \alpha , & \text{else} \end{array}\right.$$

$A^{\prime }$ is an augmented association matrix derived from the original adjacency matrix A. α serves as a hyper parameter that adjusts the margin between true labels and predicted scores. μ acts as a decay factor governing all trainable model parameters W. $\tilde{A}$ represents the predicted score matrix generated by DBGCNMDA.

### 2.6 Performance evaluation

Microbe–disease association recognition can be conceptualized as a link prediction task. Two commonly used evaluation metric, AUC and AUPR,^85^ are used to evaluate the efficacy of various methods. The higher AUC and AUPR are, the better the performance the method is.

## 3 RESULTS AND DISCUSSION

### 3.1 Performance comparison among different methods

**FIGURE 2:**
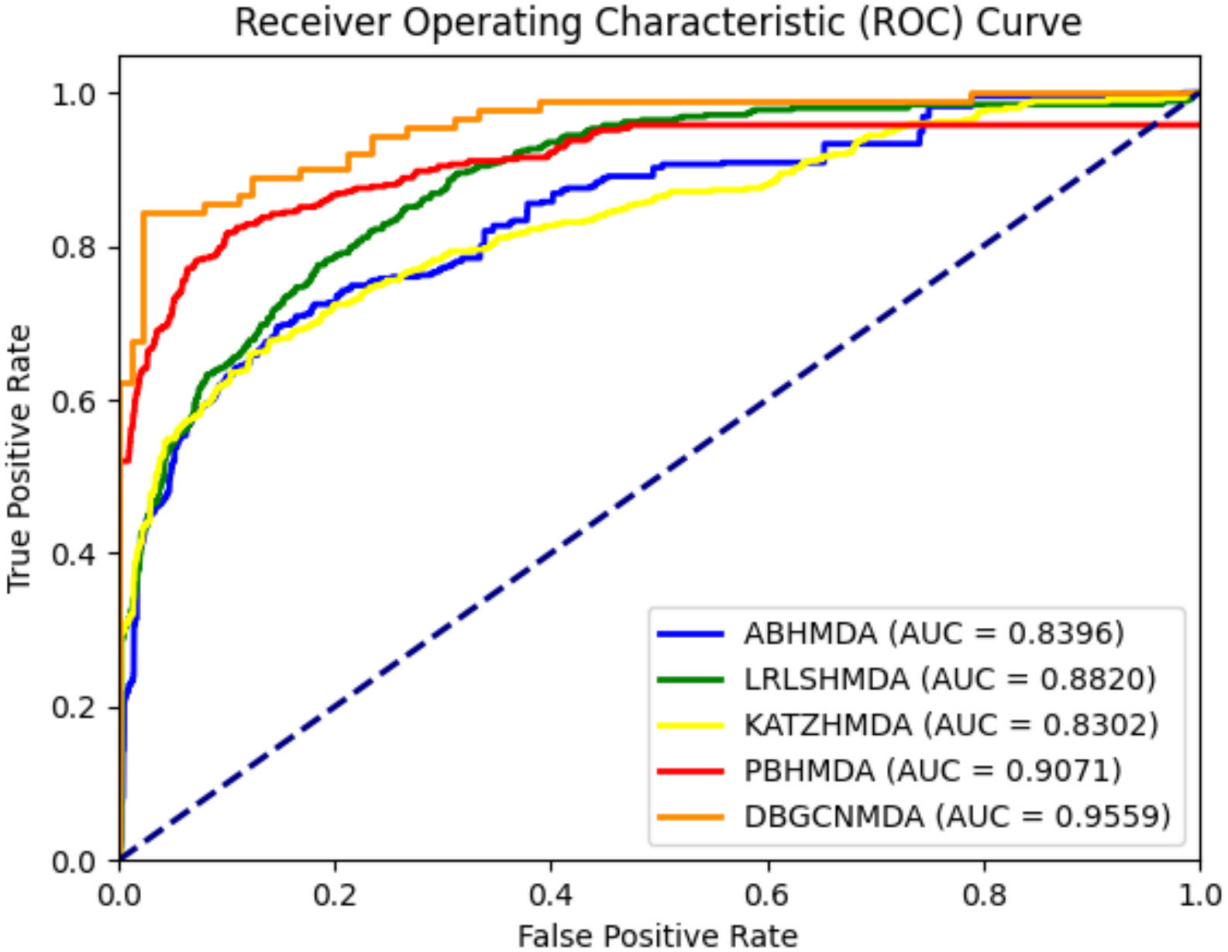
Comparison of prediction performance of DBGCNMDA with four other computational models (ABHMDA, LRLSHMDA, KATZHMDA and PBHMDA) in terms of ROC curves and AUC values. As shown in the results, the AUC of DBGCNMDA was 0.9559, which was significantly larger than that of ABHMDA (0.8396), LRLSHMDA (0.8820), KATZHMDA (0.8302) and PBHMDA (0.9071).

To evaluate the MDA prediction performance of the proposed DBGCNMDA, we compared the performance of other MDA recognition methods (ABHMDA, LRLSHMDA, KATZHMDA and PBHMDA) in predicting microbe–disease associations. ABHMDA weights multiple weak classifiers and forms a strong classifier to predict potential microbe–disease associations.^59^ LRLSHMDA is an MDA recognition algorithm based on Laplacian regularized least squares.^61^ KATZHMDA ranked all microbe–disease associations based on scores calculated from the number of walks and length between microbes and diseases.^58^ PBHMDA implemented a special deep first search algorithm to traverse all possible paths between microbes and diseases, in order to infer the most likely microbes associated with the disease.^86^ In MDA prediction, the proposed DBGCNMDA not only integrates hidden structural and attribute features, but also learns discriminative node representations through dual branch GCN, the excellent performance of DBGCNMDA is attributed to the algorithm's design, which considers two different perspectives of the receptive field from the GCN module, thereby achieving better predictive performance. The comparison of prediction performance of DBGCNMDA with four other computational models is shown in Figure 2.

### 3.2 Impact of the different components on the performance of DBGCNMDA

**FIGURE 3:**
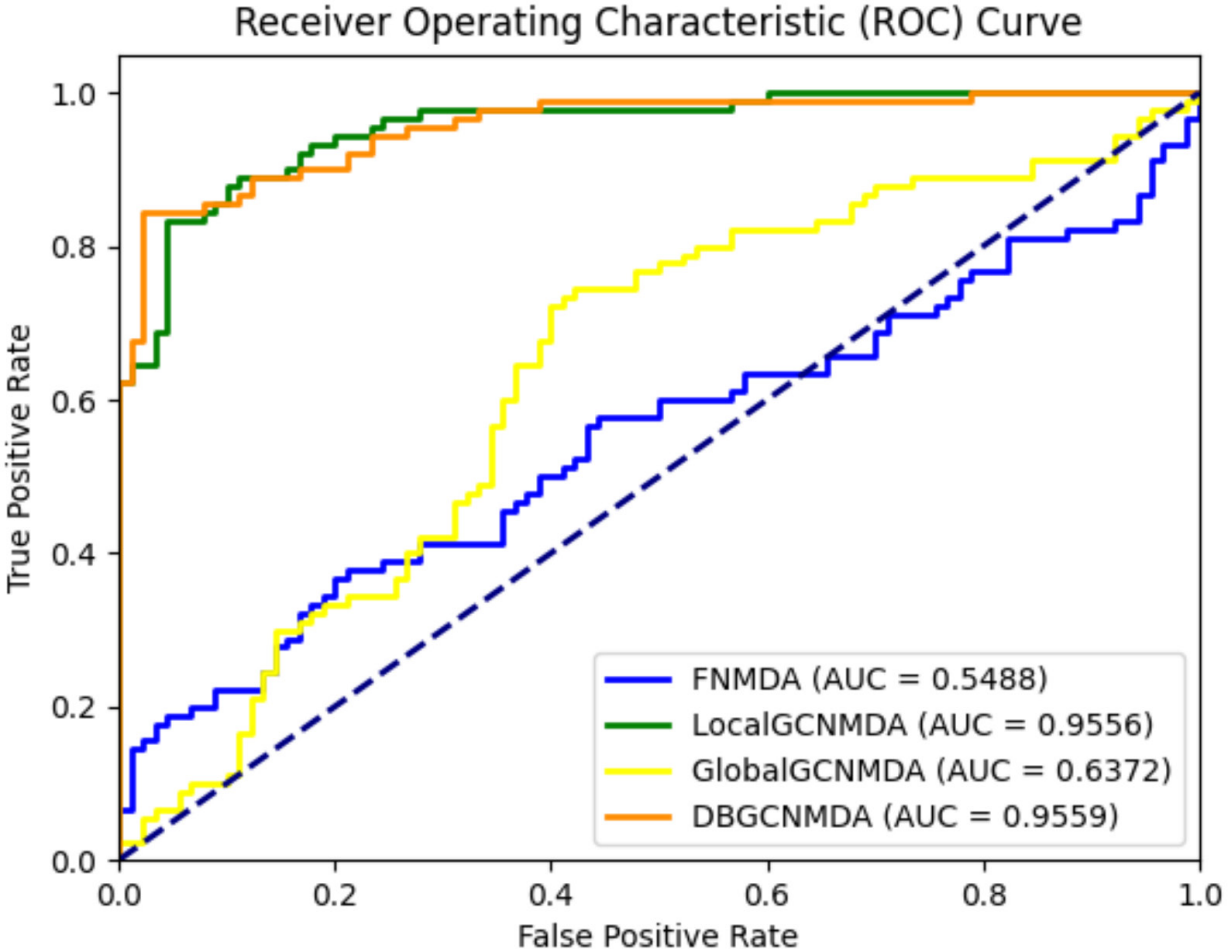
Three comparative baseline predictive factors FNMDA, GlobalGCNMDA and LocalGCNMDA in terms of ROC curves and AUC values. As shown in the results, the AUC of DBGCNMDA was 0.9559, which was significantly larger than that of FNMDA (0.5488), GlobalGCNMDA (0.6372) and LocalGCNMDA (0.9556).

There are three main components for extracting node features in DBGCNMDA: FN, GlobalGCN and LocalGCN. In order to analyse the contributions of different components in DBGCNMDA, three comparative baseline predictive factors FNMDA, GlobalGCNMDA and LocalGCNMDA were constructed. FNMDA was only constructed from fully connected networks, while GlobalGCNMDA and LocalGCNMDA were constructed from different combinations of GCN modules and fully connected networks, respectively. The predictive performance of different components is shown in Figure 3 and Table 1. The experimental results show that compared with FN, GCN contributes more to node feature extraction. The performance of the GCN module based predictor is much better than that of the FN module based predictor. At the same time, LocalGCNMDA plays a more important role than GlobalGCNMDA in capturing semantic information of two similar networks. DBGCNMDA combines three components and can achieve better performance than all other baseline prediction factors, indicating that different components can be combined to extract advanced node features.

**TABLE 1:** The performance of three comparative baseline predictive factors FNMDA, GlobalGCNMDA and LocalGCNMDA.

Predictive factors	AUC	AUPR
FNMDA	0.5488	0.6181
GlobalGCNMDA	0.6372	0.6026
LocalGCNMDA	0.9556	0.9600
**DBGCNMDA**	**0.9559**	**0.9630**

Bold values indicate that the model can achieve the best performance under this component or Number of Layers.

### 3.3 The effect of GCN layers

GCN can aggregate information from neighbouring nodes to obtain representations of microbes and diseases. To investigate the effect of GCN layers on predictive performance. We have adjusted the structure of the model, and the impact of different GCN layers on DBGCNMDA is shown in Table 2. Our research shows that the number of GCN layers has a significant impact on the predictive performance of DBGCNMDA. When DBGCNMDA does not use the GCN module (layer = 0), the prediction results of the model are approximately random guesses, and the input features are directly processed by the fully connected layer and internally generated, while using GCN can achieve better performance. This is because the lack of GCN layers cannot capture sufficient structural information, while stacked GCN layers can expand the receptive field by aggregating high‐order connected node information, thereby obtaining expressive representations. As the number of stacked GCN layers increases, the performance of DBGCNMDA's AUC and AUPR gradually improves, but when the number of layers exceeds three, the performance of DBGCNMDA decreases. This is because more stacked GCN layers may introduce more noise and irrelevant information in node representation learning, leading to excessive smoothing and performance degradation. Our experiment shows that three‐layer GCN can capture complex interaction patterns and combine node attribute features for representation learning, thereby improving prediction ability.

**TABLE 2:** The impact of GCN layers on the predictive performance of DBGCNMDA.

Number of Layers	AUC	AUPR
0	0.5842	0.5830
1	0.9502	0.9582
2	0.9468	0.9544
**3**	**0.9559**	**0.9630**
4	0.9475	0.9587

Bold values indicate that the model can achieve the best performance under this component or Number of Layers.

### 3.4 Case study

To evaluate the performance of DBGCNMDA in identifying microbes associated with known diseases, we conducted a case study. Firstly, all samples related to specific diseases were classified and literature was reviewed to confirm the established relationship between the top 10 identified microorganisms and related diseases. We selected two important diseases, inflammatory bowel disease (IBD) and rheumatoid arthritis (RA), and used DBGCNMDA to predict their associated microorganisms. Tables 3 and 4 list the top 10 microbes predicted for each disease. From Tables 3 and 4, it can be seen that out of the 20 predicted associations between microbes and diseases, 19 have been validated by experiments or biological literature.

**TABLE 3:** The 10 microbes predicted to be most likely to be associated with IBD.

Microbe	Evidence
Actinobacteria	Confirmed
*Bacteroides vulgatus*	PMID:38033588
*Clostridium coccoides*	PMID:27687331
*Clostridium difficile*	PMID:38034098
*Enterococcus*	PMID:30818368
*Haemophilus*	PMID:30685379
Lachnospiraceae	Confirmed
*Lactobacillus*	PMID:37061125
Proteobacteria	Confirmed
*Staphylococcus*	PMID:31698044

*Note*: The first column records the top 10 microbes most likely to be related with IBD, and the second column records the databases and experimental literatures in PubMed, which verify the associations between the corresponding microbe and IBD.

**TABLE 4:** The 10 microbes predicted to be most likely to be associated with RA.

Microbe	Evidence
Actinobacteria	PMID:27102666
*Clostridium coccoides*	Confirmed
*Clostridium difficile*	PMID:26908381
Enterobacteriaceae	PMID:28889208
Firmicutes	PMID:29920643
*Haemophilus*	PMID:34485325
Lachnospiraceae	PMID:35872763
*Lactobacillus*	PMID:34065638
Shuttleworthia	Unconfirmed
*Staphylococcus*	PMID:30733073

*Note*: The first column records the top 10 microbes most likely to be related with RA, and the second column records the databases and experimental literatures in PubMed, which verify the associations between the corresponding microbe and RA.

IBD refers to a group of chronic inflammatory disorders that primarily affect the gastrointestinal tract. Wu et al.'s^87^ results showed that the faecal foundation of *Bacteroides vulgaris* (*B. vulgaris*) was lower in patients with IBD than in those with IBDND. Bai et al confirmed that as a common complication of IBD, *Clostridium difficile* infection (CDI) has been shown to not only exacerbate the symptoms of IBD, but also lead to unexpected outcomes, including death.^88^ Heidari et al.^89^ identified a correlation between a reduction in the *Haemophilus* genus and an increase in BMI among IBD patients. Ni et al. demonstrated that lactobacilli could ameliorate IBD in zebrafish across various age groups by modulating the internal mucosal barrier and microbiota composition.^90^ Choi et al.^91^ evaluated the protective effect of heat killed *Enterococcus faecalis* EF‐2001 (EF‐2001) on a model of IBD. Their study strongly suggests that EF‐2001 could allocate the inflation associated with mouse IBD. The research results of Azimirad et al.^92^ indicated that patients with IBD episodes are more sensitive to co infection of *Clostridium difficile* and *Staphylococcus aureus* than to remission.

RA is a chronic autoimmune disorder that primarily affects the joints, causing inflammation, pain, stiffness and swelling. Due to the duration of the disease and the level of autoantibodies in RA patients, the diversity of gut microbiota is reduced. A taxon‐level analysis suggested an expansion of rare taxa, Actinobacteria, with a decrease in abundant taxa in patients with RA compared with controls.^93^ In a case involving a 61‐year‐old patient with *Clostridium difficile* colitis, Essrheumatoid et al. noted joint effusion following treatment, highlighting the potential contribution of *Clostridium* to reactive arthritis, despite the challenge in establishing a definitive link.^94^ Heidari et al.^95^ identified a correlation between a reduction in the *Haemophilus* genus and an increase in BMI among IBD patients. Keshteli et al. discovered that an anti‐inflammatory diet (AID) for adult UC patients led to an increase in faecal Bifidobacteriaceae, Lachnospiraceae and Ruminococcaceae.^96^ Paul et al.^97^ reviewed recent research findings to understand the overall pathogenesis of rheumatoid arthritis and the role of probiotics (especially *Lactobacillus casei* or *Lactobacillus acidophilus*) in the management of rheumatoid arthritis in clinical and preclinical studies. Goodman et al.^98^ found that the nasal carrying rate of *Staphylococcus aureus* increased in patients with rheumatoid arthritis receiving biological therapy. The above research results indicate that DBGCNMDA can discover new potential microbe–disease associations, among which unconfirmed associations can serve as candidate relationships, providing guidance for future biological experiments.

## 4 CONCLUSION

The study of the relationship between microbes and diseases is of great significance and far‐reaching impact. Understanding the relationship between microbes and diseases not only helps prevent and diagnose diseases, but also provides important theoretical support for new drug development and personalized treatment, which helps to improve human health and medical standards. In this work, we inspired GCN to effectively capture nonlinear correlation patterns in complex networks and proposed a novel computational method called DBGCNMDA for identifying microbe–disease associations. Firstly, DBGCNMDA calculates the similarity matrix between diseases and microbes by integrating their functional similarity and GAPK similarity. Then, semantic information of different biological networks is extracted through the GCN module of two different perspective fields of GCN, and finally, the score of microbe–disease association is predicted based on the extracted features. We use 5‐fold‐CV to evaluate the accuracy of the DBGCNMDA model. The results showed that the AUC and AUPR scores in the 5‐fold‐CV of the DBGCNMDA model were 0.9559 and 0.9630, respectively. Compared to previously developed computational models, the DBGCNMDA model has demonstrated a higher level of accuracy. In addition, we conducted case studies to predict a range of potential microbe–disease associations and test the predictive ability of DBGCNMDA for newly discovered microbes. The results of the case study indicate that DBGCNMDA has achieved reliable predictive performance levels. DBGCNMDA can extract semantic information from different biological networks through two differently perspective GCN modules. This approach can also be used for predicting disease–drug associations, as well as small molecule drug‐RNA associations.

Several reasons may contribute to the predictive ability of DBGCNMDA. Firstly, the data used in the model has high reliability. We evaluated the functional similarity of microbes/diseases from the perspective of biological characteristics and the GAPK similarity of microbes/diseases from the perspective of network topology. We combined functional similarity and GAPK similarity and used two types of information for microbe/disease similarity evaluation to improve MDA recognition performance. In addition, we have extended the disease nodes to address the issue of insufficient features due to low data dimensions. We use RWR to optimize the connectivity relationship between homogeneous entities, and then use the optimized similarity matrix as the initial feature matrix. In terms of network understanding, we designed dual branch GCN modules, namely GlobalGCN and LocalGCN, to fine tune node representations by introducing side information, including isomorphic neighbouring nodes. Aggregating node information in the microbe–disease interaction network through GlobalGCN. Further capture semantic information from two isomorphic and similar networks using LocalGCN.

However, certain limiting factors still exist that may affect the effectiveness of DBGCNMDA. For example, the lack of effective strategies to determine the most effective parameters of DBGCNMDA is a challenge. In addition, due to the insufficient number of training instances, the collection of confirmed microbe–disease interactions is limited, which affects the effectiveness of the model. If a broader microbe–disease relationship is empirically validated in the future, it is expected that the prognostic accuracy of DBGCNMDA will be improved. Finally, although the DBGCNMDA model can identify the association between diseases and microbes, it cannot accurately represent the complex and nonlinear interactions between microbes and diseases. Using ODE‐based theoretical modelling to study gene/protein signalling networks may be beneficial in the work of predicting microbe‐associated diseases.^99^, ^100^, ^101^, ^102^

## AUTHOR CONTRIBUTIONS

**Jing Chen:** Conceptualization (lead); methodology (lead); writing – original draft (lead). **Yongjun Zhu:** Writing – review and editing (supporting). **Qun Yuan:** Resources (supporting).

## CONFLICT OF INTEREST STATEMENT

No competing interest is declared.

## Data Availability

Data and code used in this study are available from the corresponding author upon reasonable request.
